# Prognostic value of tumour-associated neutrophils in different polarization status releasing neutrophil extracellular traps in the immunotherapy of advanced non-small cell lung cancer

**DOI:** 10.3389/fonc.2025.1705716

**Published:** 2026-01-07

**Authors:** Weiwei Hong, Xin Ye, Lin Chen, Xiangzhi Chai, Yan Yin, Zhaoqing Li, Chen Fang, Xiaoying Qian, Biao Yu, Guizhen Qin, Xinyuan Yao, Bingbiao Zhou, Chuanhong Luo, Chengsi Shu, Dengying Chen, Yong Li, Yong Wang

**Affiliations:** 1 Department of Medical Oncology, The First Affiliated Hospital of Nanchang University,Nanchang, China; 2 Medical Innovation Center, The First Affiliated Hospital of Nanchang University, Nanchang, China; 3 Department of Neurology, The Second Affiliated Hospital of Nanchang University, Nanchang, China; 4 Department of Pathology, The First Affiliated Hospital of Nanchang University, Nanchang, China

**Keywords:** neutrophil extracellular traps (NETs), non-small cell lung cancer (NSCLC), tumour-associated neutrophils (TANs), progression-free survival (PFS), overall survival (OS)

## Abstract

**Purpose:**

This study analyzed the polarization types of tumour-associated neutrophils (TANs) that release neutrophil extracellular traps (NETs), as well as the impact of neutrophil polarization on the efficacy of immunotherapy for non-small cell lung cancer (NSCLC).

**Methods:**

This study retrospectively collected clinical data and pathological samples of 115 patients with advanced NSCLC who underwent first-line immunotherapy. Multiplex immunofluorescence staining was used to assess TANs polarization status and NETs expression.

**Results:**

We found that the presence of NETs was negatively associated with tumour-associated N1 neutrophil (*P* < 0.001) but positively associated with tumour-associated N2 neutrophil (*P* < 0.001). Further analysis revealed that the NETs-low group experienced prolonged progression-free survival (PFS) (15.0 vs 9.9 months, *P* = 0.045) and overall survival (OS) (40.5 vs 22.0 months, *P* = 0.002) with first-line immunotherapy compared with the NETs-high group. We also found that there was no significant difference in the efficacy of immunotherapy between those with tumour-associated N1 neutrophils exhibiting low NETs and those exhibiting high NETs. However, patients with tumour-associated N2 neutrophils exhibiting low NETs expression experienced improved PFS (17.3 vs 9.2 months, *P* = 0.008) and OS (40.5 vs 18.3 months, *P* < 0.001) compared with that exhibiting high NETs expression. We also found that tumour-associated N2 neutrophil expressing NETs was negatively associated with CD8^+^ T cell infiltration, but positively associated with Treg cell infiltration.

**Conclusion:**

Tumour-associated N2 neutrophils in NSCLC tissues are the primary cells releasing NETs, and tumour-associated N2 neutrophils with high NETs expression are associated with an immunosuppressive tumour microenvironment, which will impact the efficacy of first-line immunotherapy in NSCLC patients.

## 1 Introduction

Lung cancer is the leading cause of cancer-related death worldwide (1). Non-small cell lung cancer (NSCLC) accounts for 80-85% of all lung cancer cases, with only a 20-30% five-year survival rate (2). Although immune checkpoint inhibitors (ICIs) are now widely used in clinical practice and have greatly improved patient survival, only 40% of NSCLC patients benefit from ICIs therapy (3). Therefore, identifying factors that can influence the response of NSCLC patients to ICIs is highly important.

The tumour immune microenvironment (TIME) is involved in the onset and development of tumours (4). Tumour-associated neutrophils (TANs) are recognised as a key component of immune infiltration within the TIME, exhibiting marked heterogeneity within tumours and exerting dual roles in both inhibiting and promoting tumour progression (5, 6). On the one hand, neutrophils play a crucial role in cancer cell elimination through mechanisms such as antibody-dependent cellular cytotoxicity (ADCC), phagocytosis, and degranulation (7–9). On the other hand, they also play an important role in promoting tumour progression. TANs facilitate cancer progression by stimulating cell proliferation and blood vessel formation (10) while also supporting tumour growth by secreting immunosuppressive cytokines (11, 12) and assisting in the immune evasion of tumour cells (13). Increasing evidence highlights the importance of TANs in the TIME of NSCLC, noting their link to tumour advancement (14).

Research has confirmed that TANs exhibit high plasticity and can alter their polarization status in response to changes in the TIME (15). On the one hand, tumour cells and immune cells within the TIME can secrete neutrophil-related chemokines, facilitating the recruitment of neutrophils and leading to changes in the number of TANs; on the other hand, cytokines within the TIME can also modify the polarization phenotypes of TANs, resulting in functional alterations in neutrophils (11, 16). TANs are categorized into two separate phenotypes: N1 neutrophils and N2 neutrophils (12). N1 neutrophils demonstrate potent antitumour effects through cytotoxic activity, ROS-mediated binding and the production of neutrophil extracellular traps (NETs) (17). Conversely, N2 neutrophils promote tumour growth by facilitating tumour angiogenesis (18) and recruiting immunosuppressive cells into the TIME (19).

NETs represent a crucial functional form of neutrophils, exhibiting dual pro-tumour and anti-tumour effects (20). Research indicates that NETs can inhibit the migration and proliferation of cultured human melanoma cells *in vitro* (21). However, in most experimental and human cancers, NETs have been reported to be closely associated with tumourigenesis and progression (20, 22–24). NETs contribute to tumour progression by trapping circulating tumour cells (25), forming a physical barrier (26) and creating a premetastatic niche (27). Furthermore, elevated NETs expression promotes the infiltration of immunosuppressive cells within the TIME (28). Researchs indicate that NETs can promote an immunosuppressive TIME by enhancing Treg cells proliferation while suppressing the cytotoxic activity of NK cells and CD8^+^ T cells (29, 30). The altered immune cell composition within the TIME of NSCLC patients, particularly marked by deficient CD8^+^ T cell infiltration and elevated Treg cell presence, significantly correlates with both immunotherapy response and disease progression (31, 32). Nevertheless, the influence of NETs on immunotherapy outcomes remains poorly understood due to insufficient investigations. Within the complex TIME, it remains uncertain whether there are discrepancies in the amount of NETs released by TANs under distinct polarization status, and whether NETs released by TANs in different polarization status exhibit varying degrees of association with the efficacy of immunotherapy for NSCLC.

In light of these recent findings, it appears that both TANs and the NETs they released may influence the prognosis of the NSCLC patients. Our research focused on analyzing the prognostic value of TANs in different polarization status releasing NETs in the immunotherapy of advanced NSCLC patients receiving first-line immunotherapy. Additionally, to explore the potential mechanisms underlying the impact of these factors on the efficacy of immunotherapy for NSCLC, we conducted correlation analyses among the expression levels of NETs, the polarization status of TANs, and the infiltration of immune cells.

## 2 Materials and methods

### 2.1 Study population

This retrospective study enrolled 115 advanced NSCLC patients treated with first-line immune checkpoint inhibitors at the First Affiliated Hospital of Nanchang University from May 2019 to May 2021. The inclusion criteria were as follows: (a) histopathological/cytological confirmation of advanced NSCLC, (b) completion of ≥2 cycle of ICI treatment, and (c) the presence of at least one measurable lesion according to RECIST v1.1. Pretreatment tissue samples and clinical data were obtained for all participants.

### 2.2 Immunohistochemical staining

The primary antibodies used for immunohistochemistry included CD8 (RMA-0514; Maxim) and Foxp3 (BS-0269R; Bioss) antibodies. Paraffin-embedded (FFPE) samples were sectioned into 4 μm thick tissue slices and dewaxed using xylene (100%, 95%, 90%) and gradient alcohols (85%, 80%, 70%). Following antigen retrieval, microwave the samples at 100°C for 20 minutes. After peroxidase blockade (3% H_2_O_2_) and nonspecific site blocking (5% BSA), primary antibody incubation was performed at 4°C overnight. Two blinded pathologists independently evaluated immunoreactivity, with final quantification representing triplicate field averages.

### 2.3 Multiplex immunofluorescence staining

Multiplex immunofluorescence staining was performed on FFPE samples. The primary antibodies used included CD11b (ab133357; Abcam), CD206 (ab64693; Abcam), MPO (ab208670; Abcam), and citH3 (NB100-57135; Novus Biologicals). Following primary antibody incubation (1 hour, RT), the samples were incubated with HRP-conjugated secondary antibodies (Akoya Biosciences) and counterstained with DAPI (20 min). Fluorescence imaging was performed using both widefield (Nikon Eclipse E100) and confocal (Nikon Eclipse C1) microscopy systems. Myeloperoxidase (MPO) and CD11b immunofluorescence co-staining were defined as neutrophil (17). Immunofluorescence co-staining with DNA, MPO, and citrullinated histone H3 (citH3) visualises NETs formation (33–35). CD206 expression effectively distinguishes between N1 and N2 neutrophils (17). Two blinded pathologists independently analysed all multispectral images without access to clinical data. The quantity of TANs was evaluated by taking the average count from three independent observations under a ×400 high-power microscope (HPF). Optimal cut-off thresholds were established through X-tile software (Yale University), a specialized bioinformatics platform designed for biomarker assessment and outcome-driven cut-off point determination. Indicators equal to or below these thresholds are classified as low expression, while those above the thresholds are classified as high expression.

### 2.4 Survival follow-up

Patients underwent regular monitoring following the first dose of ICIs. The objective response rate (ORR) refers to the proportion of patients achieving complete response (CR) and a partial response (PR), while the disease control rate (DCR) represents the combined proportion of patients with PR, CR and stable disease (SD). We defined progression-free survival (PFS) as the interval between the start of treatment and either disease progression or death. Overall survival (OS) was measured from the first ICIs dose to either death or final observation.

### 2.5 Statistical analysis

In this research, statistical analyses were conducted using SPSS 24.0. Continuous variables were compared using appropriate parametric (t test) or nonparametric (Mann-Whitney U) tests on the basis of data distribution. Connections between NETs, TANs, clinicopathological features, and immune cell presence were assessed using the Pearson chi-square test or Fisher’s exact test. To analyse associations between two continuous variables, we used Spearman’s method or the Mann-Whitney U test. Differences in PFS and OS among the participants were assessed via the log-rank test with Kaplan-Meier curves. Univariate and multivariate Cox proportional hazards models were used to identify independent prognostic factors. A threshold for statistical significance was set at a two-tailed *P* value under 0.05.

## 3 Results

### 3.1 Demographic and clinicopathological characteristics

The initial clinicopathological profiles of the enrolled patients in the NSCLC cohort treated with first-line immune checkpoint inhibitors are detailed in **Table 1**. Twenty-nine patients were younger than 60 years (25.2%), 99 patients were male (86.1%), and 40.0% patients were non-smokers. For 106 patients (92.2%), the Eastern Cooperative Oncology Group Performance Status (ECOG PS) analysis revealed that approximately 64.3% of these individuals had adenocarcinoma, whereas 35.7% had squamous cell carcinoma. In terms of TNM staging, 52% of patients with NSCLC were classified as having advanced T stages (T3 and T4), and 78.3% were categorized into severe N stages (N2 and N3). Forty percent of the individuals were stage III, and 60% were stage IV (AJCC 8), with 74.8% experiencing metastases at two or more locations.

**Table 1:** Patient characteristics included in the study.

Variables	N	Percent
Age (years)		
<60	29	25.2%
≥60	86	74.8%
Sex		
Male	99	86.1%
Female	16	13.9%
Smoking status		
Yes	69	60.0%
No	46	40.0%
ECOG PS		
0-1	106	92.2%
2	9	7.8%
Histology		
Adenocarcinoma	74	64.3%
Squamous carcinoma	41	35.7%
Tumour stage		
T1-2	55	48.0%
T3-4	60	52.0%
Lymph node metastasis		
N0-1	25	21.7%
N2-3	90	78.3%
TNM stage		
III	46	40.0%
IV	69	60.0%
Number of metastatic sites		
0-1	86	25.2%
≥2	29	74.8%
Type of therapy		
only immunotherapy	7	6.1%
with one another treatment	92	80.0%
with two another treatment	16	13.9%
NETs area, cut off (mix-max)	1083(15-18248)	
Tumour-associated total neutrophils, cut off (mix-max)	18(4-47)	
Tumour-associated N1 neutrophils, cut off (mix-max)	8(1-40)	
Tumour-associated N2 neutrophils, cut off (mix-max)	6 (1-21)	
The ratio of N1/N2, cut off (mix-max)	1.67(0.20-21.00)	
Tumour-associated N1^+^ neutrophils, cut off (mix-max)	3(1-18)	
Tumour-associated N2^+^ neutrophils, cut off (mix-max)	3 (1-13)	
The ratio of N^+^/N, cut off (mix-max)	0.5(0-1.00)	
Tumour-infiltrating CD8^+^ T cells, cut off (mix-max)	35(0-301)	
Tumour-infiltrating Treg cells, cut off (mix-max)	62(0-439)	
The ratio of CD8^+^ T/Treg, cut off (mix-max)	0.54(0-142.75)	

ECOG PS, Eastern Cooperative Oncology Group Performance Status.

### 3.2 Relationship between NETs expressing and TANs polarization state

**Figure 1:**
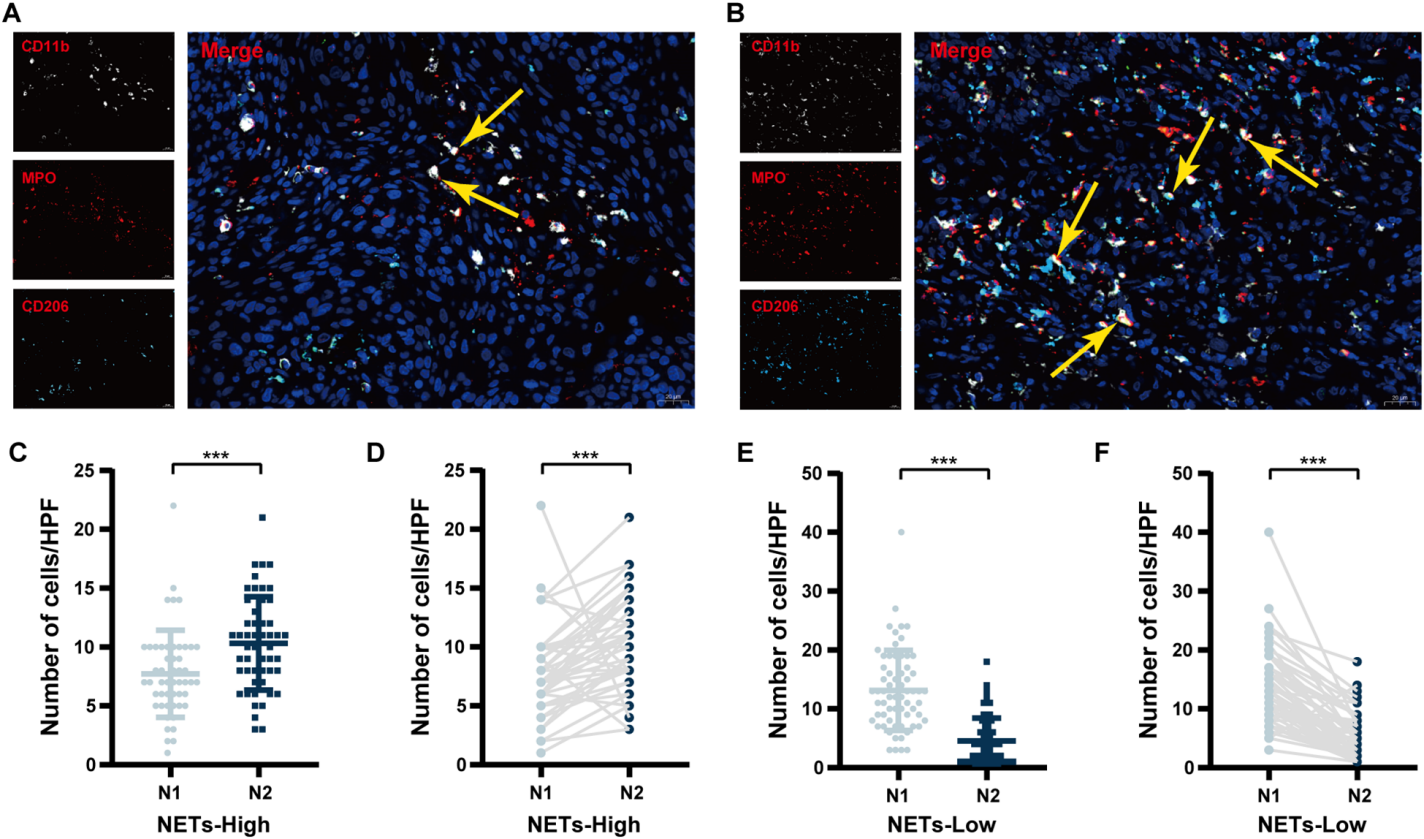
Comparison of TANs polarization status between NETs-high and NETs-low groups. **(A)** Representative immunofluorescence images of tumour-associated N1 neutrophils; **(B)** Representative immunofluorescence images of tumour-associated N2 neutrophils; **(C)** A comparison of the numbers of tumour-associated N1 and N2 neutrophils in the NETs-high group by unpaired analysis; **(D)** A comparison of the numbers of tumour-associated N1 and N2 neutrophils in the NETs-high group by paired analysis; **(E)** A comparison of the numbers of tumour-associated N1 and N2 neutrophils in the NETs-low group by unpaired analysis; **(F)** A comparison of the numbers of tumour-associated N1 and N2 neutrophils in the NETs-low group by paired analysis. IF, immunofluorescence; TANs, tumour-associated neutrophils; NSCLC, non-small cell lung cancer; HPF, high-power field; TANs, tumour-associated neutrophils; NETs, neutrophil extracellular traps. ^***^P < 0.001.

As shown in **Figure 1** and **Supplementary Figure 1**, tumour-associated N1 neutrophils were defined as MPO^+^CD11b^+^CD206^-^ cells, denoted as N1 (**Figure 1A**). Tumour-associated N2 neutrophils were defined as MPO^+^CD11b^+^CD206^+^ cells (17) and denoted N2 (**Figure 1B**). NETs were detected via combined staining for MPO and citH3 (**Supplementary Figure 1A**). N1^+^ neutrophils were defined as tumour-associated N1 neutrophils that release NETs, specifically MPO^+^CD11b^+^CD206^-^citH3^+^ cells (**Supplementary Figure 1B**). N2^+^ neutrophils were defined as tumour-associated N2 neutrophils that release NETs, specifically MPO^+^CD11b^+^CD206^+^citH3^+^ cells (**Supplementary Figure 1C**). N^+^ neutrophils include N1^+^ and N2^+^ neutrophils.

Based on X-tile software analysis, the ideal thresholds for NETs expression area, N, N1 and N2 neutrophils, as well as the N1/N2 ratio, N1^+^ neutrophils, N2^+^ neutrophils, and the N^+^/N ratio, were determined to be 1083um (2), 18/HPF, 8/HPF, 6/HPF, 1.67, 3/HPF, 3/HPF, and 0.50, respectively (**Table 1**). Unpaired analysis revealed that the mffedian number of tumour-associated N2 neutrophils in the NETs-high group reached 10/HPF, exceeding the number of tumour-associated N1 neutrophils, which was 7/HPF (*P* < 0.001, **Figure 1C**). Pairwise analysis revealed notable differences between tumour-associated N1 and N2 neutrophils (*P* < 0.001, **Figure 1D**). In contrast, within the NETs-low group, the median tumour-associated N2 neutrophil count was 4/HPF, which was significantly lower than the tumour-associated N1 neutrophil count of 14.5/HPF (*P* < 0.001, **Figure 1E**). Similar results emerged from paired analysis (*P* < 0.001, **Figure 1F**).

**Figure 2:**
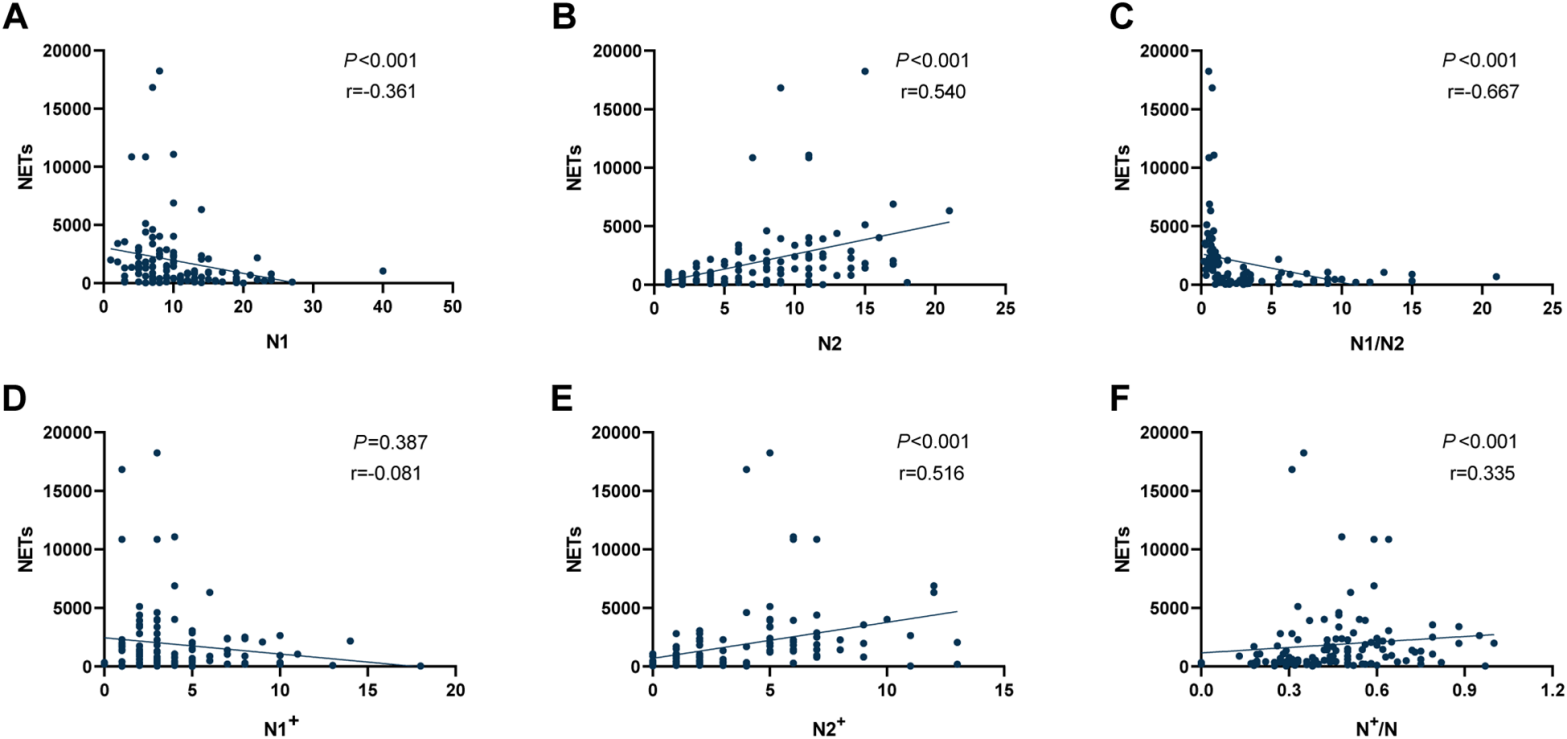
Correlation analysis between NETs expression levels and different types of TANs in the TIME of NSCLC. **(A)** Correlation between NETs and tumour-associated N1 neutrophils; **(B)** Correlation between NETs and tumour-associated N2 neutrophils; **(C)** Correlation between NETs and the ratio of N1/N2; **(D)** Correlation between NETs and tumour-associated N1^+^ neutrophils; **(E)** Correlation between NETs and tumour-associated N2^+^ neutrophils; **(F)** Correlation between NETs and the ratio of N^+^/N. NETs, neutrophil extracellular traps; TANs, tumour-associated neutrophils; TIME, tumour immune microenvironment; NSCLC, non-small cell lung cancer.

In addition, we conducted a correlation analysis between NETs levels and the polarization status of TANs, as shown in **Figure 2**. NETs expression exhibited a negative correlation with tumour-associated N1 neutrophil counts (r=-0.361, *P* < 0.001; **Figure 2A**), although this association was not robust. However, NETs expression showed a significant positive correlation with tumour-associated N2 neutrophil counts (r = 0.540, *P* < 0.001; **Figure 2B**), and a higher N1/N2 ratio correlated with lower NETs expression (r=-0.667, *P* < 0.001; **Figure 2C**). NETs levels showed no statistically significant relationship with tumour-associated N1^+^ neutrophils (r=-0.081, *P* = 0.387; **Figure 2D**), but exhibited a significant positive correlation with tumour-associated N2^+^ neutrophils (r=0.516, *P* < 0.001; **Figure 2E**). A positive correlation trend was also observed with N^+^/N (r=0.335, *P* < 0.001; **Figure 2F**).

### 3.3 Associations between NETs or TANs and clinicopathological characteristics

We evaluated the relationships among TANs and clinicopathological factors, as detailed in **Table 2**. Our analysis revealed no significant differences between the N-low and N-high groups, N1-low and N1-high groups or between the N1/N2-low and N1/N2-high groups when variables such as age, sex, smoking habits, ECOG PS, pathological type, tumour stage, lymph node involvement, TNM classification, number of metastatic sites, and treatment options were considered, confirming the comparability between the two groups. However, a significantly greater proportion of smokers was observed in the N2-low subgroup than in the N2-high subgroup (70.0% vs 50.9%, *P* = 0.048), with no other substantial differences noted. The number of patients in the CD8^+^ T-low and CD8^+^ T-high groups, as well as those in the Treg-low and Treg-high groups, showed no significant difference between the N-low and N-high groups in the TIME (*P* = 0.105; *P* = 0.338). Furthermore, there were no significant differences between the N1-low and N1-high groups concerning patients in the CD8^+^ T-low and CD8^+^ T-high groups, as well as those in the Treg-low and Treg-high groups in the TIME (*P* = 0.324; *P* = 0.432). However, in the N2-low group, we observed a higher proportion of patients exhibiting elevated CD8^+^ T-cell infiltration (*P* = 0.030), whereas in the N2-high group, more patients demonstrated reduced CD8^+^ T-cell infiltration (*P* < 0.001).

**Table 2:** Relationships between NETs, tumour-associated N1 neutrophils, N2 neutrophils, N1/N2 ratio and clinicopathological characteristics.

Variables	Tumour associated total neutrophils				Tumour associated N1 neutrophils			Tumour associated N2 neutrophils			N1/N2		
	Low		High	*P* value	Low	High	*P* value	Low	High	*P* value	Low	High	*P* value
Age (years)													
<60	16	13		0.616	10	19	0.258	17	12	0.308	11	18	0.119
≥60	52	34			40	46		41	45		47	39	
Sex													
Male	61	38		0.177	42	57	0.571	51	48	0.564	47	52	0.114
Female	7	9			8	8		7	9		11	5	
Smoking status													
Yes	40	29		0.757	27	42	0.249	40	29	**0.048**	30	39	0.068
No	28	18			23	23		18	28		28	18	
ECOG PS													
0-1	62	44		0.736	47	59	0.792	54	52	0.743	53	53	1.000
2	6	3			3	6		4	5		5	4	
Histology													
Adenocarcinoma	25	16		0.764	20	21	0.393	18	23	0.297	23	18	0.366
Squamous carcinoma	43	31			30	74		40	34		35	39	
Tumour stage													
T1-2	31	24		0.563	25	30	0.682	27	28	0.783	27	28	0.783
T3-4	37	23			25	35		31	29		31	29	
Lymph node metastasis													
N0-1	14	11		0.719	12	13	0.606	13	12	0.860	12	13	0.783
N2-3	54	36			38	52		45	45		46	44	
TNM stage													
III	30	16		0.278	21	25	0.701	26	20	0.286	22	24	0.648
IV	38	31			29	40		32	37		36	33	
Number of metastatic sites													
0-1	52	34		0.616	38	48	0.792	43	43	0.872	45	41	0.485
≥2	16	13			12	17		15	14		13	16	
Type of therapy													
only immunotherapy	5	2		0.740	1	6	0.272	4	3	0.935	4	3	0.935
with one another treatment	53	39			42	50		46	46		46	46	
with two another treatment	10	6			7	9		58	57		8	8	
NETs													
≤1083um^2^	42	22		0.112	17	47	**<0.001**	48	16	**<0.001**	10	54	**<0.001**
>1083um^2^	26	25			33	18		10	41		48	3	
Tumour associated N1 neutrophils													
≤8/HPF	–	–		**-**	–	–	–	23	27	0.404	38	12	**<0.001**
>8/HPF	–	–			–	–		35	30		20	45	
Tumour associated N2 neutrophils													
≤6/HPF	–	–		**-**	23	35	0.404	–	–	–	12	46	**<0.001**
>6/HPF	–	–			27	30		–	–		46	11	
Tumour associated N1/N2 neutrophils													
≤1.67	31	27		0.211	38	20	**<0.001**	12	46	**<0.001**	**-**	**-**	**-**
>1.67	37	20			12	45		46	11		–	–	
Tumour associated N^+^/N neutrophils													
≤0.05	45	29		0.622	31	43	0.645	41	33	0.152	31	43	**0.014**
>0.05	23	18			19	22		17	24		27	14	
CD8^+^ T cells infiltrating													
≤35/HPF	33	30		0.105	30	33	0.324	26	37	**0.030**	40	23	**0.002**
>35/HPF	35	17			20	32		32	20		18	34	
Treg cells infiltrating													
≤62/HPF	38	22		0.338	24	36	0.432	39	21	**<0.001**	20	40	**<0.001**
>62/HPF	30	25			26	29		19	36		38	17	
CD8^+^/Treg													
≤0.54	31	25		0.423	27	29	0.318	21	35	**0.007**	37	19	**0.001**
>0.54	37	22			23	36		37	22		21	38	

ECOG PS, Eastern Cooperative Oncology Group Performance Status; HPF, high-power fields. The bolded values represent results with a P-value less than 0.05.

In addition, we analysed the relationships between polarization status of TANs expressing NETs and immune cell infiltration, as shown in **Supplementary Table 1**. We found no significant differences in age, sex, smoking status, ECOG PS, pathological type, tumour stage, lymph node metastasis, TNM stage, number of metastatic sites, or treatment type between the NETs-low and NETs-high groups, N1^+^-low and N1^+^-high groups, the N2^+^-low and N2^+^-high groups, or the N^+^/N-low and N^+^/N-high groups, indicating their comparability. Additionally, we assessed the contrast in neutrophil and immune cell infiltration patterns between patients with high and low NET counts. Notably, the NETs-low group had a significantly higher number of patients with high tumor-associated N1 neutrophil (47 vs 18, *P* < 0.001) and low Treg cell (21 vs 34, *P* < 0.001) infiltration compared to the NETs-high group. Additionally, compared with the NETs-high group, the NETs-low group also showed a trend toward an increased number of patients with high CD8^+^ T cell infiltration (34 vs 18, *P* = 0.056).

Among patients in the N1^+^-low and N1^+^-high groups, there were no significant differences in the number of patients with CD8^+^ T-low and CD8^+^ T-high, Treg-low and Treg-high, or CD8^+^ T/Treg-low and CD8^+^ T/Treg-high ratios (*P* = 0.344; *P* = 0.424; *P* = 0.529). In contrast, among patients in the N2^+^-low and N2^+^-high groups, 36 and 16 patients were identified as CD8^+^ T-high, respectively (*P* = 0.005). There were 21 patients in the N2^+^-low group and 34 in the N2^+^-high group (*P* < 0.001). Similarly, 41 and 18 CD8^+^ T/Treg-high patients were included in the N2^+^-low and N2^+^-high groups, respectively (*P* = 0.001). Additionally, our findings revealed a notably greater number of CD8^+^ T/Treg-high patients in the N^+^/N-low group than in the N^+^/N-high group, with values of 46 and 13, respectively (*P* = 0.002).

### 3.4 The correlation among the expression levels of NETs, the polarization status of TANs, and the efficacy of immunotherapy

As shown in **Supplementary Figure 2**, the N2-low group demonstrated a notably greater ORR and DCR than the N2-high group did (ORR: 65.5% vs 43.9%, *P* = 0.020; DCR: 94.8% vs 84.2%, *P* = 0.063). In contrast, the N1/N2-low group had a significantly lower ORR than N1/N2-high group (44.8% vs 64.9%, *P* = 0.030), while no significant difference in DCR was observed between the N1/N2-low and N1/N2-high groups (86.2% vs 93.0%, *P* = 0.235). There were no significant differences in ORR or DCR between the N-low and N-high groups, the N1-low and N1-high groups, the N2-low and N2-high groups, the NETs-low and NETs-high groups, the N1^+^-low and N1^+^-high groups, the N2^+^-low and N2^+^-high groups, or the N^+^/N-low and N^+^/N-high groups.

**Figure 3:**
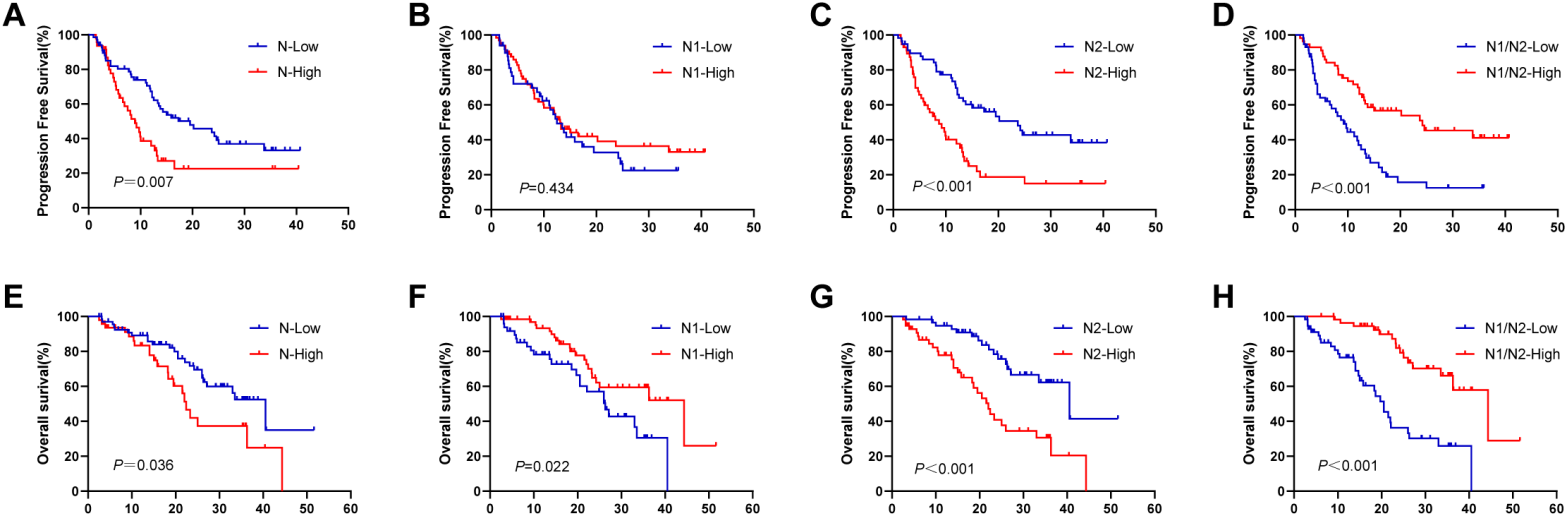
Survival analysis in terms of TANs. **(A, E)** KaplanMeier plots for PFS and OS according to the different number of tumour-associated total neutrophils; **(B, F)** KaplanMeier plots for PFS and OS according to the different number of tumour-associated N1 neutrophils; **(C, G)** KaplanMeier plots for PFS and OS according to the different number of tumour-associated N2 neutrophils; **(D, H)** KaplanMeier plots for PFS and OS according to the different ratio of N1/N2. TANs, tumour-associated neutrophils; PFS, progression-free survival; OS, overall survival.

Further examination of the survival data revealed that patients in the N-low group demonstrated significantly prolonged PFS and OS compared with those in the N-high group (PFS: 19.5 vs 8.9 months, *P* = 0.007, **Figure 3A**; OS: 40.5 vs 22.4 months, *P* = 0.036, **Figure 3E**). No notable difference was found in PFS between the N1-high and N1-low groups, with durations of 12.5 months and 13.1 months, respectively (*P* = 0.434, **Figure 3B**). However, OS was greater in the N1-high group at 44.3 months than in the N1-low group at 26.3 months (*P* = 0.022, **Figure 3F**). In contrast, the N2-low group achieved significantly greater PFS and OS than the N2-high group did, with PFS recorded at 23.7 months compared with 8.7 months (*P* < 0.001, **Figure 3C**) and OS at 40.5 months compared with 22.0 months (*P* < 0.001, **Figure 3G**). Compared with the N1/N2-high group, the combined N1/N2-low group had markedly lower PFS and OS times (9.2 vs 24.2 months, *P* < 0.001, **Figure 3D**; 20.5 vs 44.3 months, *P* < 0.001, **Figure 3H**).

**Figure 4:**
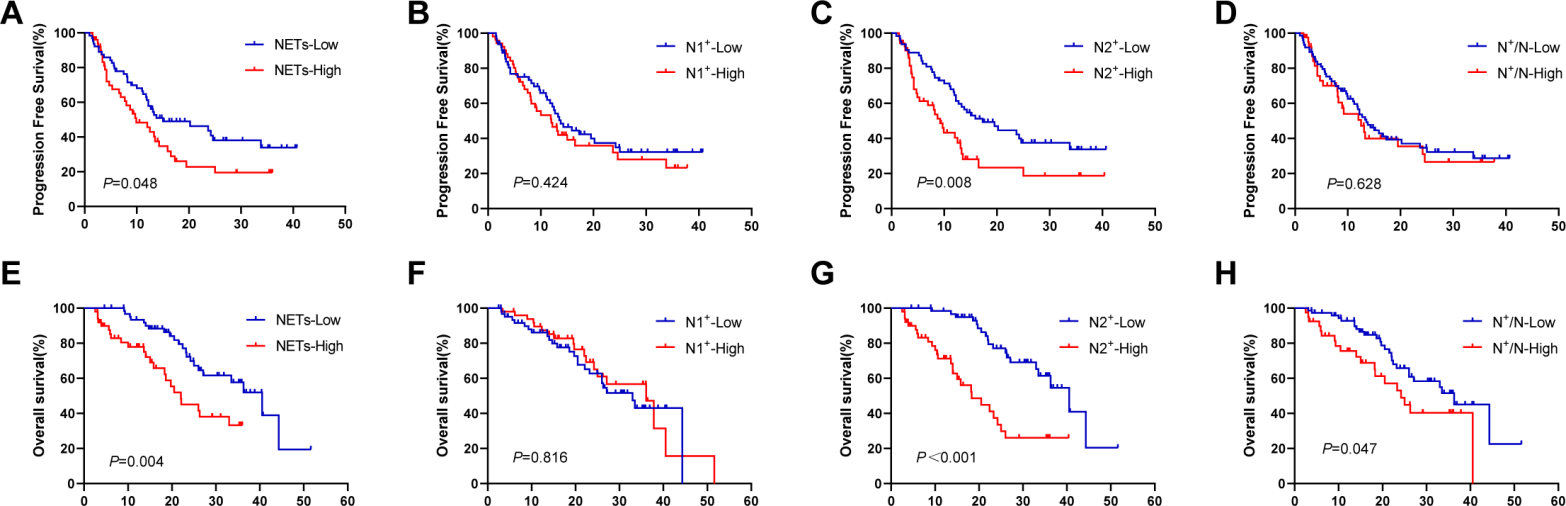
Survival analysis in terms of TANs expressing NETs. **(A, E)** KaplanMeier plots for PFS and OS according to the different area of NETs; **(B, F)** KaplanMeier plots for PFS and OS according to the different number of tumour-associated N1^+^ neutrophils; **(C, G)** KaplanMeier plots for PFS and OS according to the different number of tumour-associated N2^+^ neutrophils; **(D, H)** KaplanMeier plots for PFS and OS according to the different ratio of N^+^/N. TANs, tumour-associated neutrophils; NETs, neutrophil extracellular traps; PFS, progression-free survival; OS, overall survival.

We further investigated the relationship between NETs and the efficacy of immunotherapy, and found that the NETs-low group presented longer PFS and OS than the NETs-high group did, with PFS values of 15.0 months compared with 9.9 months (*P* = 0.048, **Figure 4A**) and OS values of 40.5 months compared with 22.0 months (*P* = 0.004, **Figure 4E**). The N1^+^-low group demonstrated comparable PFS and OS to the N1^+^-high group (PFS: 13.8 vs 12.0 months, *P* = 0.424; **Figure 4B**; OS: 33.0 vs 36.3 months, *P* = 0.816; **Figure 4F**). In contrast, the N2^+^-low group exhibited significantly greater PFS and OS than the N2^+^-high group did (PFS: 17.3 vs 9.2 months, *P* = 0.008; **Figure 4C**; OS: 40.5 vs 18.3 months, *P* < 0.001; **Figure 4G**). Compared with the N^+^/N-high group, the N^+^/N-low group had no significant improvement in PFS (12.5 vs 13.5 months, *P* = 0.628; **Figure 4D**) but had a significantly longer OS (36.3 vs 24.2 months, *P* = 0.047; **Figure 4H**).

### 3.5 Relationship between immune cell infiltration in the TIME and the efficacy of immunotherapy in patients with NSCLC

To improve our comprehension of the immunosuppressive mechanisms within the TIME of NSCLC patients, we reanalyzed previously examined pathological tissue samples. The representative images of IHC staining for CD8^+^ T cells and Tregs are shown in **Supplementary Figure 3**. Using Xtil software, the optimal cut-off values for CD8^+^ T cells, Treg cells, and the ratio of CD8^+^ T cells to Treg cells (CD8^+^ T/Treg) in the TIME were determined to be 35/HPF, 62/HPF, and 0.54, respectively (**Table 1**). As shown in **Supplementary Figure 4**, the ORRs for the CD8^+^ T-low and CD8^+^ T-high groups, the Treg-low and Treg-high groups, and the CD8^+^ T/Treg-low and CD8^+^ T/Treg-high groups were 49.2% and 61.5%, 61.7% and 47.3%, 48.2% and 61.0%, respectively, with no statistically significant differences observed (*P* = 0.186, *P* = 0.121, *P* = 0.168, **Supplementary Figure 4A**). However, we found that the DCRs for the CD8^+^ T-high, Treg-low, and CD8^+^ T/Treg-high groups were 96.2%, 95.0%, and 98.3%, respectively, which were significantly greater than those for the CD8^+^ T-low (84.1%), Treg-high (83.6%), and CD8^+^ T/Treg-low (80.4%) groups (*P* = 0.036, *P* = 0.046, *P* = 0.002, **Supplementary Figure 4B**). Additionally, we analysed the impact of immune cell infiltration on survival. As depicted in **Supplementary Figure S5**, we found that the PFS times for the CD8^+^ T-high group and Treg-low group were 14.3 months and 20.2 months, respectively, which were significantly longer than those for the CD8^+^ T-low group (11.8 months) and Treg-high group (8.0 months) (*P* = 0.023, **Supplementary Figure 5A**; *P* < 0.001; **Supplementary Figure 5B**). The OS times for the CD8^+^ T-high group and Treg-low group were 36.3 months and 44.3 months, respectively, which were significantly longer than those for the CD8^+^ T-low group (22.4 months) and Treg-high group (20.5 months) (*P* = 0.005, **Supplementary Figure 5D**; *P* < 0.001, **Supplementary Figure 5E**). Furthermore, our observations revealed that the PFS times for the CD8^+^ T/Treg-low group and CD8^+^ T/Treg-high group were 8.1 months and 16.5 months, respectively, and the OS times were 20.5 months and 44.3 months, with significantly prolonged survival for the CD8^+^ T/Treg-high group (*P* < 0.001, **Supplementary Figure 5C**; *P* < 0.001, **Supplementary Figure 5F**).

### 3.6 Relationships between NETs or TANs polarization status and immune cell infiltration

**Figure 5:**
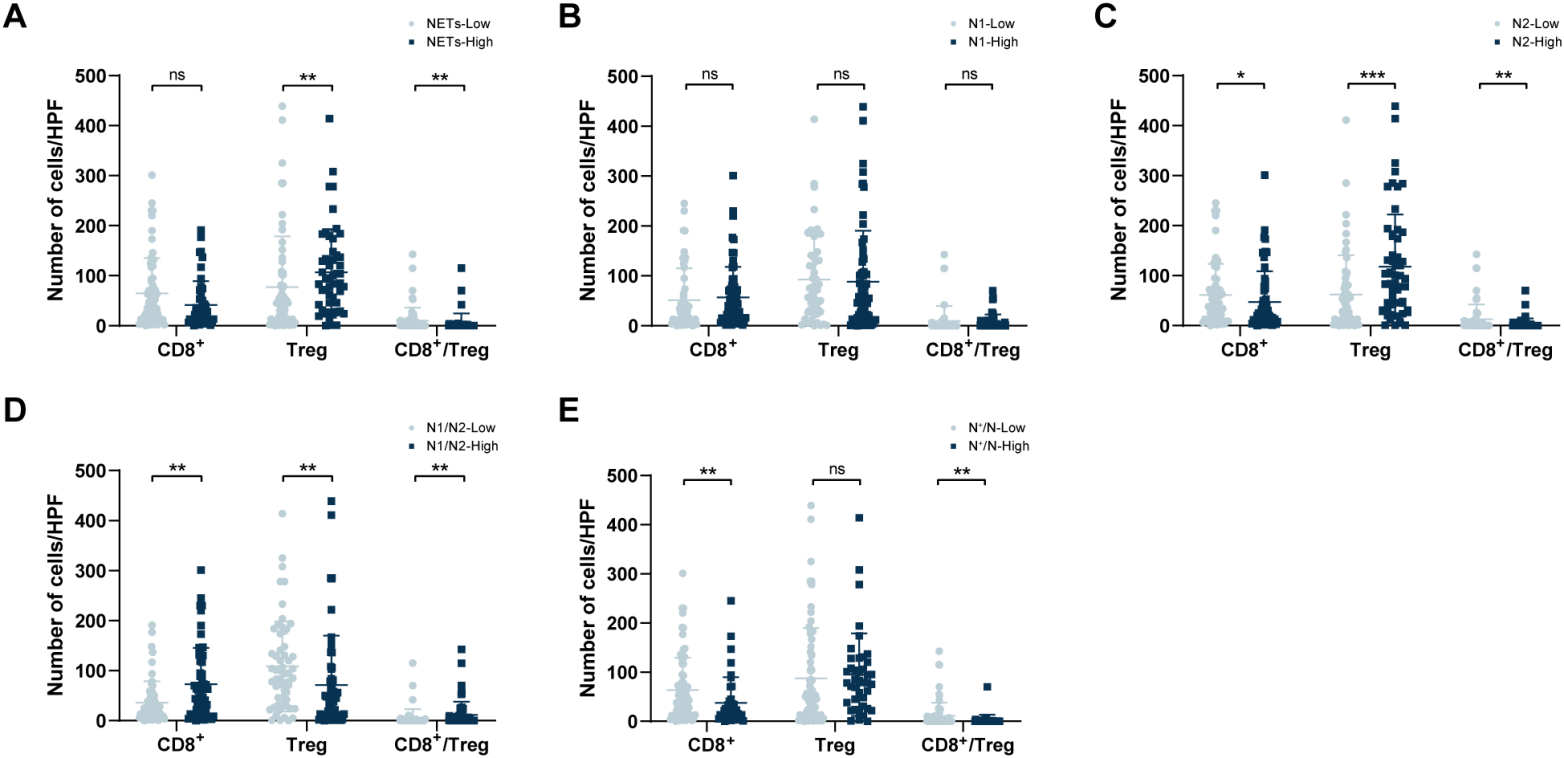
Correlation between TANs and infiltration of intratumoral CD8^+^ T cells and Tregs in the TIME. **(A)** Comparison of tumour-infiltrating CD8^+^ T cell and Treg numbers within different NETs groups; **(B)** Comparison of tumour-infiltrating CD8^+^ T cell and Treg numbers within different N1 groups. **(C)** Comparison of tumour-infiltrating CD8^+^ T cell and Treg numbers within different N2 groups; **(D)** Comparison of tumour-infiltrating CD8^+^ T cell and Treg numbers within different N1/N2 ratio groups; **(E)** Comparison of tumour-infiltrating CD8^+^ T cell and Treg numbers within different N^+^/N ratio groups. TANs, tumour-associated neutrophils; TIME, tumour immune microenvironment. ^*^P < 0.05, ^**^P < 0.01, ^***^P < 0.001.

In the NETs-low group, the median values of CD8^+^ T cells, Treg cells, and the ratio of CD8^+^ T/Treg in the TIME were 40.5/HPF, 41.5/HPF, and 0.785, respectively. In contrast, in the NETs-high group, these values were 26.0/HPF (*P* = 0.087), 85.0/HPF (*P* = 0.003), and 0.260 (*P* = 0.012) (**Figure 5A**). Between the N1-low and N1-high groups, the median values of CD8^+^ T cells, Treg cells, and the CD8^+^ T/Treg ratio in the TIME were 26.0/HPF compared with 35.0/HPF, 70.5/HPF compared with 54.0/HPF, and 0.26 compared with 0.60, respectively, but these differences were not statistically significant (*P* = 0.200, *P* = 0.370, *P* = 0.127) (**Figure 5B**). Within the TIME, significant differences in the infiltration of CD8^+^ T cells and Treg cells were observed between the N2-low group and the N2-high group (CD8^+^ T: 40.5/HPF vs 22.0/HPF, *P* = 0.043; Treg: 37.0/HPF vs 94.0/HPF, *P* < 0.001; CD8^+^ T/Treg ratio: 0.91 vs 0.24, *P=*0.003; **Figure 5C**). Compared with those in the N1/N2-high group, the CD8^+^ T cell counts and CD8^+^ T/Treg ratio were markedly lower in the N1/N2-low group (CD8^+^ T: 22.5/HPF vs 47.0/HPF, *P* = 0.003; CD8^+^ T/Treg ratio: 0.22 vs 1.15, *P=*0.001), but Treg cell counts were significantly greater in the N1/N2-low group(89.5/HPF vs 38.0/HPF, *P* = 0.001) (**Figure 5D**). Additionally, the median values of CD8^+^ T cells and the CD8^+^ T/Treg ratio in the low N^+^/N group were 41.5/HPF and 0.77, respectively, which were greater than those in the high N^+^/N2 group (22.0/HPF, *P* = 0.008; 0.21, *P* = 0.001), whereas the number of Treg cells was lower (46.0/HPF vs 79.0/HPF, *P* = 0.128) (**Figure 5E**).

As shown in **Supplementary Figure 6**, there was an inverse relationship between the number of CD8^+^ T cells and both the number of NETs and the presence of N2 cells (r=-0.196, *P* = 0.036; **Supplementary Figure 6A**; r=-0.222, *P* = 0.017; **Supplementary Figure 6C**). In contrast, the number of CD8^+^ T cells was positively correlated with the N1 neutrophil count and the N1/N2 ratio, with correlation coefficients of 0.186 and 0.306, respectively (*P* = 0.046, **Supplementary Figure 6B**; *P* < 0.001, **Supplementary Figure 6D**). The number of Treg cells was significantly positively associated with the number of NETs and the number of N2 neutrophils (*p* = 0.016, **Supplementary Figure 6E**; *P* = 0.001, **Supplementary Figure 6G**), whereas no noteworthy correlation was found with the number of N1 neutrophils (*P* = 0.229, **Supplementary Figure 6F**). A negative correlation was identified between Treg cells and the N1/N2 ratio (*P* = 0.001, **Supplementary Figure 6H**). Higher CD8^+^ T/Treg ratios were associated with increased N1 neutrophil counts and N1/N2 ratios (*P* = 0.018, **Supplementary Figure 6J**; *P* < 0.001, **Supplementary Figure 6L**), as well as elevated NETs expression and N2 neutrophil counts (*P* = 0.012, **Supplementary Figure 6I**; *P* = 0.002, **Supplementary Figure 6K**).

Additionally, we analysed the correlations between immune cell infiltration and the polarization status of NETs-expressing TANs. Our analysis revealed that CD8^+^ T cells were not significantly correlated with N1^+^ neutrophil counts (r=-0.008, *P* = 0.928; **Supplementary Figure 7A**). However, there was a negative correlation between CD8^+^ T cells and both N2^+^ neutrophils and the N^+^/N ratio, with correlation coefficients of -0.287 and -0.253, respectively (*P* = 0.002, **Supplementary Figure 7B**; *P* = 0.006, **Supplementary Figure 7C**). Moreover, the Treg cell count and the CD8^+^ T/Treg ratio were not significantly correlated with the N1^+^ neutrophil count (r=0.050, *P* = 0.600; **Supplementary Figure 7D**; r=-0.015, *P* = 0.871; **Supplementary Figure 7G**). However, Tregs displayed positive correlations with both N2^+^ neutrophil counts (r=0.326, *P* < 0.001; **Supplementary Figure 7E**; r=0.195, *P* = 0.037; **Supplementary Figure 7F**), whereas CD8^+^ T/Treg ratio was negatively correlated with both parameters (r=-0.203, *P* = 0.030; **Supplementary Figure 7H**; r=-0.322, *P* < 0.001; **Supplementary Figure 7I**).

### 3.7 Analysis of prognostic factors in first-line immunotherapy-treated NSCLC patients

Univariate Cox analysis indicated that the ECOG PS (*P* < 0.001) and pathological type (*P* = 0.045) were significantly correlated with PFS. Furthermore, T stage (*P* = 0.019, *P* = 0.012), tumour-associated N2 neutrophils (*P* < 0.001, *P* < 0.001), tumour-associated N2^+^ neutrophils (*P* = 0.009, *P* < 0.001), CD8^+^ T cells (*P* = 0.025, *P* = 0.007), and Treg cells (*P* < 0.001, *P* < 0.001) were significantly associated with both PFS and OS. Additionally, the NETs expression level (*P* = 0.005) and number of tumour-associated N1 neutrophils (*P* = 0.025) were identified as risk factors influencing OS (**Table 3**). Subsequent multivariate Cox analysis revealed that pathological type (*P* = 0.028, HR = 1.778, CI = 1.064-2.970), T stage (*P* = 0.019, HR = 1.845, CI = 1.108-3.074), tumour-associated N2 neutrophils (*P* = 0.018, HR = 2.477, CI = 1.170-5.242), and Treg cells (*P* = 0.033, HR = 1.782, CI = 1.048-3.030) were independent predictors of PFS. Concurrently, T stage (*P* = 0.027, HR = 2.123, CI = 1.088-4.142), tumour-associated N1 neutrophils (*P* = 0.004, HR = 0.356, CI = 0.178-0.714), tumour-associated N2^+^ neutrophils (*P* = 0.007, HR = 3.565, CI = 1.408-9.028), and Treg cells (*P* = 0.004, HR = 2.672, CI = 1.362-5.241) were identified as independent prognostic factors for OS, as shown in **Table 3**.

**Table 3:** Univariate and multivariate analysis of prognostic factors associated with progression-free survival and overall survival.

Variables	PFS			OS		
	Univariate *P* value	Multivariate HR (95%CI)	Multivariate *P* value	Univariate *P* value	Multivariate HR (95%CI)	Multivariate *P* value
Age (years)						
<60/≥60	0.416		NA	0.137		NA
Sex						
Male/Female	0.138		NA	0.629		NA
Smoking status						
Yes/No	0.095		NA	0.798		NA
ECOG						
0-1/2	<**0.001**	2.272 (0.965, 5.348)	0.060	0.157		NA
Histology						
Adenocarcinoma/Squamous carcinoma	**0.045**	1.778 (1.064, 2.970)	**0.028**	0.983		NA
Tumour stage						
T1-2/T3-4	**0.019**	1.845 (1.108, 3.074)	**0.019**	**0.012**	2.123 (1.088, 4.142)	0.027
Lymph node metastasis						
N0-1/N2-3	0.819		NA	0.659		NA
TNM stage						
III/IV	0.134		NA	0.722		NA
Number of metastatic sites						
0-1/≥2	0.388		NA	0.911		NA
Type of therapy						
only immunotherapy/ with one another reatment/ with two another treatment	0.209		NA	0.477		NA
NETs						
≤1083um^2^/>1083um^2^	0.051		NA	**0.005**	0.592 (0.264, 1.328)	0.203
Tumour associated N1 neutrophils						
≤8/>8	0.436		NA	**0.025**	0.356 (0.178, 0.714)	**0.004**
Tumour associated N2 neutrophils						
≤6/>6	**<0.001**	2.477 (1.170, 5.242)	**0.018**	**<0.001**	2.109 (0.932, 4.771)	0.073
Tumour associated N1^+^ neutrophils						
≤3/>3	0.426		NA	0.816		NA
Tumour associated N2^+^ neutrophils						
≤3/>3	**0.009**	0.995 (0.480, 2.059)	0.988	**<0.001**	3.565 (1.408, 9.028)	**0.007**
CD8^+^ T cells infiltrating						
≤35/>35	**0.025**	0.739 (0.426, 1.281)	0.282	**0.007**	0.653 (0.336, 1.271)	0.210
Treg cells infiltrating						
≤62/>62	**<0.001**	1.782 (1.048, 3.030)	**0.033**	**<0.001**	2.672 (1.362, 5.241)	**0.004**

ECOG PS, Eastern Cooperative Oncology Group Performance Status; PFS, progression-free survival; OS, overall survival. The bolded values represent results with a P-value less than 0.05.

## 4 Discussion

Our research first investigated the relationship between the levels of NETs and the polarization status of TANs within the TIME of NSCLC, as well as their interaction with immune cell infiltration to evaluate their impact on the efficacy of immunotherapy in advanced NSCLC patients. Our findings indicated that the expression of NETs is primarily associated with the quantity of tumour-associated N2 neutrophils in the TIME of NSCLC, and found that a greater infiltration of tumour-associated N2 neutrophils with high NETs expression correlates with poorer immunotherapy outcomes in NSCLC patients. This may be related to the role of tumour-associated N2 neutrophils expressing NETs in promoting an immunosuppressive environment in NSCLC, thereby facilitating immune escape.

Neutrophils are the most abundant immune cells in the TIME (36) and are closely associated with tumorigenesis and progression. An increasing number of studies indicate that the presence of TANs in malignant tissues is strongly linked to the severity and progression of the tumour, metastasis, and unfavourable survival rates among cancer patients (37–39). TANs contribute to the spread of cancer cells to distant sites through multiple processes, such as creating an immunosuppressive environment around the tumour, aiding in the establishment of conditions favourable for metastasis, and supporting the colonization of tumour cells (40, 41).Research has indicated that neutrophils play a role in facilitating tumour evasion by the immune system (13). Our study also found that the total number of TANs were associated with poor prognosis in NSCLC patients. These findings align with previous research indicating that elevated TANs counts correlates with poorer clinical outcomes (17).

In recent years, despite advances in TANs research, controversy persists regarding their markers. Studies indicate that multiple markers, including myeloperoxidase (MPO), CD66b, and CD15, can be employed to identify TANs (42). CD11b on the surface of neutrophils plays a crucial role in cell adhesion and phagocytosis (43). Consequently, Chen et al. successfully identified TANs through the co-localisation phenomenon of MPO and CD11b (17). NETs, as products of neutrophil activation, are reticular structures composed of DNA as a scaffold, interspersed with citrullinated histone H3 (citH3), neutrophil elastase (NE), myeloperoxidase (MPO), and other components. Multiple studies indicate that immunofluorescence co-staining with DNA, MPO, and citH3 effectively visualises NETs formation (33–35). Consequently, we employed immunofluorescence co-staining with MPO, CD11b and citH3 to mark TANs associated with NETs formation. Fridlender et al. categorised TANs into N1 and N2 subtypes, drawing upon tumour-associated macrophage (TAMs) classification schemes (44). However, the markers distinguishing N1 and N2 neutrophils remain to be definitively identified. Research indicates that CD206 exhibits characteristics in neutrophils similar to those observed in macrophages (45, 46). Chen et al. utilised single-cell RNA sequencing analysis to classify neutrophils into CD206^-^ and CD206^+^ subtypes. They observed that the functional characteristics of these neutrophil types align with previously reported tumour-associated N1 and N2 neutrophil functions, concluding that CD206 expression effectively distinguishes between N1 and N2 neutrophils (17). Therefore, we have employed CD206 as the marker for distinguishing between N1 and N2 neutrophils in our study.

Furthermore, our analysis of the correlation between neutrophil polarisation status and prognosis revealed that NSCLC patients exhibiting elevated tumour-associated N2 neutrophil infiltration experienced poorer ORR, PFS and OS following initial ICIs therapy, whereas those with increased tumour-associated N1 neutrophil infiltration showed notably longer survival durations posttreatment. One potential explanation for this phenomenon is that N2 neutrophils have an extended lifespan and reduced toxicity relative to N1 neutrophils, in addition to having roles that support blood vessel formation, dampen immune responses, and enhance tumour development (18, 47). These findings reinforce the literature indicating that N1 neutrophils primarily exert antitumour effects, whereas N2 neutrophils predominantly support tumour progression (12). Therefore, we hypothesize that tumour-associated N2 neutrophils may play a dominant role in the TIME of NSCLC patients treated with ICIs.

Our study revealed a substantial connection between NETs expression and TANs polarization status. In the TIME of advanced NSCLC patients, those with elevated NETs expression presented a notable increase in N2 neutrophils. Conversely, patients with low NETs expression presented a marked increase in N1 neutrophils. This further confirms previous findings that N2 neutrophils increase during tumour progression and are the primary TANs that produce NETs (48). NETs exhibite both anti-tumour and pro-tumour effects (20, 49), with their specific effects primarily determined by their interaction with the TIME (21). Liu et al. discovered that NETs inhibit bladder cancer growth and dissemination by promoting T-cell and monocyte-macrophage recruitment (50). However, in most experimental and human cancers, NETs have been reported to be closely associated with tumourigenesis and progression (34, 51, 52). Moreover, emerging data indicate a connection between NETs formation and evasion of antitumour immune responses, underscoring the potential of suppressing NETs levels to increase tumour susceptibility to ICIs therapy (53). Jian et al. reported that serum NETs are a reliable predictor of the effectiveness of PD-1 inhibitor therapy in patients with NSCLC (54). Our research has found that NETs concentrations in tumour tissue are also associated with the efficacy of immunotherapy in NSCLC patients.

Moreover, we observed that patients with N2 neutrophils exhibiting high NETs expression demonstrated poorer prognosis compared to those with N2 patients showing low NETs expression, whereas the level of NETs expression in N1 neutrophils showed no significant impact on prognosis. This suggests that NETs produced by these two neutrophil subtypes may possess distinct physiological functions. The polarisation process of TANs depends on different stimulatory factors. Research indicates that interferon-β (IFN-β) and tumour necrosis factor-α (TNF-α) induce TANs polarization toward the N1 phenotype, while cytokines such as TGF-β, IL-6 and G-CSF promote polarization toward the N2 phenotype (44, 55). The phenotypic shift accompanying TANs polarisation is accompanied by alterations in the protein secretome and functional profile (56). NETs are products of neutrophil activation, with their formation process and composition being highly complex (57). Currently, the factors determining NETs function remain unclear. Therefore, whether alterations in the polarization status of TANs lead to functional differences in the NETs they produce warrants further investigation.

Our study revealed that the levels of CD8^+^ T cells and Treg cells in the TIME significantly impact the effectiveness of immunotherapy. Elevated concentrations of CD8^+^ T cells and reduced Treg cell numbers within tumour tissues are correlated with prolonged PFS and OS, which aligns with earlier study results (31, 32). Research indicates that interactions between various cell types within the TIME can help cancer cells escape immune detection, thus increasing tumour growth and spread (49). We explored the connection between TANs and the presence of CD8^+^ T lymphocytes along with Treg cells in the tumour environment. Our results indicated that individuals exhibiting elevated quantities of N2 neutrophils or a diminished N1/N2 ratio presented reduced infiltration of CD8^+^ T cells and an increased presence of Treg cells in tumour areas. Conversely, a greater number of N1 neutrophils was positively correlated with increased CD8^+^ T-cells infiltration and a greater ratio of CD8^+^ T/Treg cells. This phenomenon may be attributed to interactions among tumour-infiltrating immune cells (17, 49). Research indicates that N1 neutrophils are recognized for releasing proinflammatory and immune-enhancing cytokines that aid in attracting CD8^+^ T cells (58). In contrast, N2 neutrophils are known to release high levels of ARG-1, which suppresses T-cell function within tumours (59).

Additionally, the connection between NETs expression and the infiltration of immune cells was examined. Our findings indicate that increased NETs expression correlates negatively with the number of CD8^+^ T cells infiltration, whilst correlating positively with the number of Treg cells infiltration. A higher ratio of CD8^+^ T/Treg corresponded to lower NETs expression and a reduced N^+^/N ratio. These findings are consistent with the literature suggesting a connection between NETs formation and immune evasion in tumours (53). Research indicates that NETs formation may contribute to immune evasion by influencing PD-1/PD-L1 expression, promoting Treg cell differentiation, inhibiting the quantity and function of CD8^+^ T cells, and creating physical barriers to exert immunosuppressive effects (49). Thus, modulating NETs expression might increase tumour susceptibility to ICIs, offering a potential approach to increase the efficacy of immunotherapy in medical practice.

Our research investigated the associations between NETs and TANs polarization status in the TIME of NSCLC patients. Our findings revealed high NET expression is primarily significantly associated with an increased number of tumour-associated N2 neutrophils. We also found that patients with a high proportion of tumour-associated N2 neutrophils exhibited poorer survival outcomes. Further research has revealed that the NET expression level in tumour-associated N1 neutrophils has no significant impact on prognosis, whereas patients with a higher count of tumour-associated N2 neutrophils exhibiting high NETs expression have a poorer prognosis. Furthermore, we analysed the relationships among TANs polarization status, NETs, CD8^+^ T cells, and Treg infiltration in tumour tissues, which warrants further investigation into potential mechanisms. Subsequently, we pinpointed that tumour-associated N2 neutrophil expressing NETs was negatively associated with CD8^+^ T cell infiltration, but positively associated with Treg cell infiltration. Additional research is needed to investigate how the polarization status of TANs impacts NETs and to explore the connection between NETs and the effectiveness of immunotherapy.

This study has several limitations that warrant consideration. Primarily, its retrospective design introduces inherent constraints. Furthermore, the single-centre nature of this retrospective analysis potentially limits the generalizability of the findings. To address these limitations, future research should prioritize large-scale, prospective, multicentre cohort studies. Secondly, the current methods for classifying N1/N2 neutrophils remain unclear, and the reliability of CD206-based markers for distinguishing N1 and N2 neutrophil subtypes requires more rigorous experimental validation. Thirdly, this study has not elucidated the specific mechanisms underlying the polarisation of TANs towards N1 or N2 neutrophils. Fourthly, whether the mechanisms underlying NETs production following neutrophil polarisation and the functional properties of such NETs differ requires further experimental validation. Fifthly, the article only discusses the relationship between neutrophil polarisation status and the quantity of infiltrating immune cells, without further investigating whether this affects T cell function, such as cytokine/chemokine marker detection or T cell suppression assays.

## 5 Conclusions

In summary, our findings indicate that NETs expression correlate with the polarisation status of TANs, and this association is linked to the infiltration of immunosuppressive cells within the TIME. Tumour-associated N2 neutrophils expressing high levels of NETs potentially exerting a dominant immunosuppressive influence in the NSCLC microenvironment. This reflects the role of TANs in immune evasion. Further investigations into the effects of NETs and neutrophils polarization status on various immune cell populations will significantly advance our understanding of TANs-mediated mechanisms of immunotherapy resistance, ultimately contributing to the development of more effective and targeted therapeutic interventions.

## Data Availability

The raw data supporting the conclusions of this article will be made available by the authors, without undue reservation.
