# Supplementary material for: Prognostic value of tumour-associated neutrophils in different polarization status releasing neutrophil extracellular traps in the immunotherapy of advanced non-small cell lung cancer
Source: Front Oncol. 2026 Jan 7;15:1705716. doi: 10.3389/fonc.2025.1705716 (PMC12819214; doi:10.3389/fonc.2025.1705716)
Supplement: Supplementary file 1 [file DataSheet1.docx]

**Supplemental table 1 Relationships between tumour-associated N1^+^, N2^+^ and N^+^/N ratio and clinicopathological characteristics**

| **Variables** | **NETs** | | | | **Tumour associated N1^+^**  **neutrophils** | | | | **Tumour associated N2^+^**  **neutrophils** | | | | | **N^+^/N** | | |
| --- | --- | --- | --- | --- | --- | --- | --- | --- | --- | --- | --- | --- | --- | --- | --- | --- |
|  | Low | | High | *P* value | Low | High | *P* value | | Low | | High | P value | | Low | High | *P* value |
| Age (years) |  |  | |  |  |  |  | |  | |  |  | |  |  |  |
| <60 | 19 | 10 | | 0.216 | 15 | 14 | 0.702 | | 17 | | 12 | 0.631 | | 23 | 6 | 0.052 |
| ≥60 | 45 | 41 | |  | 48 | 38 |  | | 46 | | 40 |  | | 51 | 35 |  |
| Sex |  |  | |  |  |  |  | |  |  | | |  |  |  |  |
| Male | 58 | 41 | | 0.115 | 55 | 44 | 0.679 | | 55 | 44 | | | 0.679 | 63 | 36 | 0.692 |
| Female | 6 | 10 | |  | 8 | 8 |  | | 8 | 8 | | |  | 11 | 5 |  |
| Smoking status |  |  | |  |  |  |  | |  |  | | |  |  |  |  |
| Yes | 41 | 28 | | 0.319 | 35 | 34 | 0.284 | | 40 | 29 | | | 0.400 | 45 | 24 | 0.812 |
| No | 23 | 23 | |  | 28 | 18 |  | | 23 | 23 | | |  | 29 | 17 |  |
| ECOG PS |  |  | |  |  |  |  | |  |  | | |  |  |  |  |
| 0-1 | 57 | 49 | | 0.295 | 60 | 46 | 0.296 | | 57 | 49 | | | 0.509 | 68 | 38 | 1.000 |
| 2 | 7 | 2 | |  | 3 | 6 |  | | 6 | 3 | | |  | 6 | 3 |  |
| Histology |  |  | |  |  |  |  | |  |  | | |  |  |  |  |
| Adenocarcinoma | 23 | 18 | | 0.943 | 23 | 18 | 0.833 | | 24 | 17 | | | 0.547 | 29 | 12 | 0.287 |
| Squamous carcinoma | 41 | 33 | |  | 40 | 34 |  | | 39 | 35 | | |  | 45 | 29 |  |
| Tumour stage |  |  | |  |  |  |  | |  |  | | |  |  |  |  |
| T1-2 | 31 | 24 | | 0.883 | 33 | 22 | 0.282 | | 30 | 25 | | | 0.961 | 39 | 16 | 0.160 |
| T3-4 | 33 | 27 | |  | 30 | 30 |  | | 33 | 27 | | |  | 35 | 25 |  |
| Lymph node metastasis |  |  | |  |  |  |  | |  |  | | |  |  |  |  |
| N0-1 | 15 | 10 | | 0.621 | 13 | 12 | 0.752 | | 13 | 12 | | | 0.752 | 15 | 10 | 0.608 |
| N2-3 | 49 | 41 | |  | 50 | 40 |  | | 50 | 40 | | |  | 59 | 31 |  |
| TNM stage |  |  | |  |  |  |  | |  |  | | |  |  |  |  |
| III | 26 | 20 | | 0.878 | 26 | 20 | 0.760 | | 27 | 19 | | | 0.491 | 31 | 15 | 0.578 |
| IV | 38 | 31 | |  | 37 | 32 | |  | 36 | 33 | | |  | 43 | 26 |  |
| Number of metastatic sites |  |  | |  |  |  | |  |  |  | | |  |  |  |  |
| 0-1 | 46 | 40 | | 0.421 | 47 | 39 | | 0.961 | 46 | 40 | | | 0.631 | 55 | 31 | 0.879 |
| ≥2 | 18 | 11 | |  | 16 | 13 | |  | 17 | 12 | | |  | 19 | 10 |  |
| Type of therapy |  |  | |  |  |  | |  |  |  | | |  |  |  |  |
| only immunotherapy | 4 | 3 | | 0.886 | 4 | 3 | | 0.825 | 4 | 3 | | | 0.825 | 3 | 4 | 0.452 |
| with one another treatment | 52 | 40 | |  | 49 | 43 | |  | 49 | 43 | | |  | 61 | 31 |  |
| with two another treatment | 8 | 8 | |  | 10 | 6 | |  | 10 | 6 | | |  | 10 | 6 |  |
| NETs |  |  | |  |  |  | |  |  |  | | |  |  |  |  |
| ≤1083um^2^ | - | - | | - | 30 | 34 | | 0.056 | 53 | 11 | | | **<0.001** | 48 | 16 | **0.008** |
| >1083um^2^ | - | - | |  | 33 | 18 | |  | 10 | 41 | | |  | 26 | 25 |  |
| Tumour associated N1 neutrophils |  |  | |  |  |  | |  |  |  | | |  |  |  |  |
| ≤8/HPF | 17 | 33 | | **<0.001** | 44 | 6 | | **<0.001** | 23 | 27 | | | 0.097 | 31 | 19 | 0.645 |
| >8/HPF | 47 | 18 | |  | 19 | 46 | |  | 40 | 25 | | |  | 43 | 22 |  |
| Tumour associated N2 neutrophils |  |  | |  |  |  | |  |  |  | | |  |  |  |  |
| ≤6/HPF | 40 | 10 | | **<0.001** | 32 | 26 | | 0.932 | 52 | 6 | | | **<0.001** | 41 | 17 | 0.152 |
| >6/HPF | 16 | 41 | |  | 31 | 26 | |  | 11 | 46 | | |  | 33 | 24 |  |
| Tumour associated N1/N2 neutrophils |  |  | |  |  |  | |  |  |  | | |  |  |  |  |
| ≤1.67 | 10 | 48 | | **<0.001** | 39 | 19 | | **0.007** | 15 | 43 | | | **<0.001** | 31 | 27 | **0.014** |
| >1.67 | 54 | 3 | |  | 24 | 33 | |  | 48 | 9 | | |  | 43 | 14 |  |
| Tumour associated N^+^/N neutrophils |  |  | |  |  |  | |  |  |  | | |  |  |  |  |
| ≤0.05 | 48 | 26 | | **0.008** | 49 | 25 | | **<0.001** | 51 | 23 | | | **<0.001** | **-** | **-** | **-** |
| >0.05 | 16 | 25 | |  | 14 | 27 | |  | 12 | 29 | | |  | - | - |  |
| CD8^+^ T cells infiltrating |  |  | |  |  |  | |  |  |  | | |  |  |  |  |
| ≤35/HPF | 30 | 33 | | 0.056 | 32 | 31 | | 0.344 | 27 | 36 | | | **0.005** | 33 | 30 | **0.003** |
| >35/HPF | 34 | 18 | |  | 31 | 21 | |  | 36 | 16 | | |  | 41 | 11 |  |
| Treg cells infiltrating |  |  | |  |  |  | |  |  |  | | |  |  |  |  |
| ≤62/HPF | 43 | 17 | | **<0.001** | 35 | 25 | | 0.424 | 42 | 18 | | | **<0.001** | 45 | 15 | **0.013** |
| >62/HPF | 21 | 34 | |  | 28 | 27 | |  | 21 | 34 | | |  | 29 | 26 |  |
| CD8^+^/Treg |  |  | |  |  |  | |  |  |  | | |  |  |  |  |
| ≤0.54 | 26 | 30 | | 0.052 | 29 | 27 | | 0.529 | 22 | 34 | | | **0.001** | 28 | 28 | **0.002** |
| >0.54 | 38 | 21 | |  | 34 | 25 | |  | 41 | 18 | | |  | 46 | 13 |  |

ECOG PS, Eastern Cooperative Oncology Group Performance Status; HPF: high-power fields.

**Supplementary Figures**


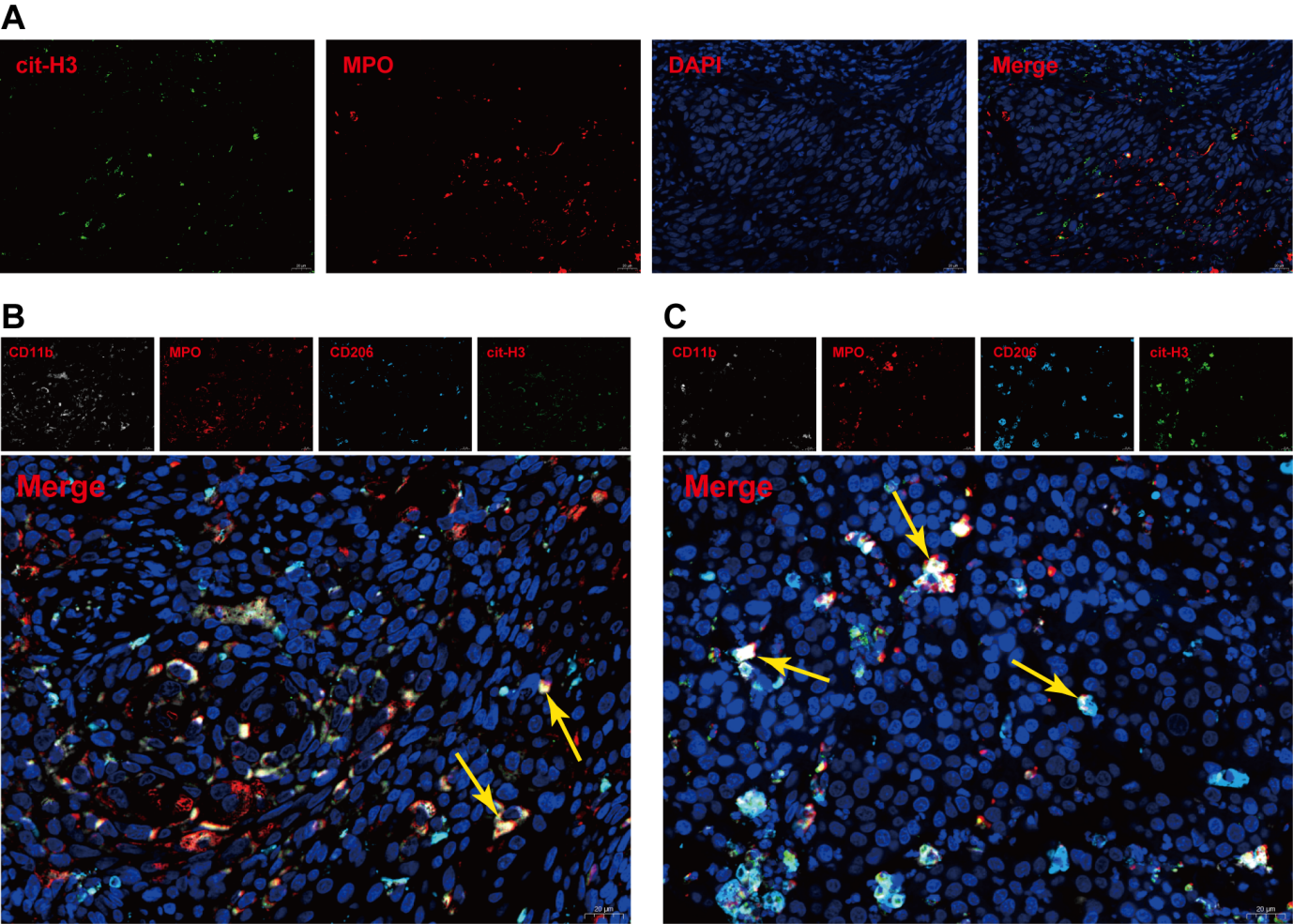


**sFigure 1 Representative images of IF staining for TANs expressing NETs in the TIME of NSCLC patients.** (A) Representative immunofluorescence images of NETs; (B) Representative immunofluorescence images of tumour-associated N1^+^ neutrophils; (C) Representative immunofluorescence images of tumour-associated N2^+^ neutrophils. IF, immunofluorescence; NETs, neutrophil extracellular traps; TANs, tumour-associated neutrophils; TIME, tumour immune microenvironment; NSCLC, non-small cell lung cancer.


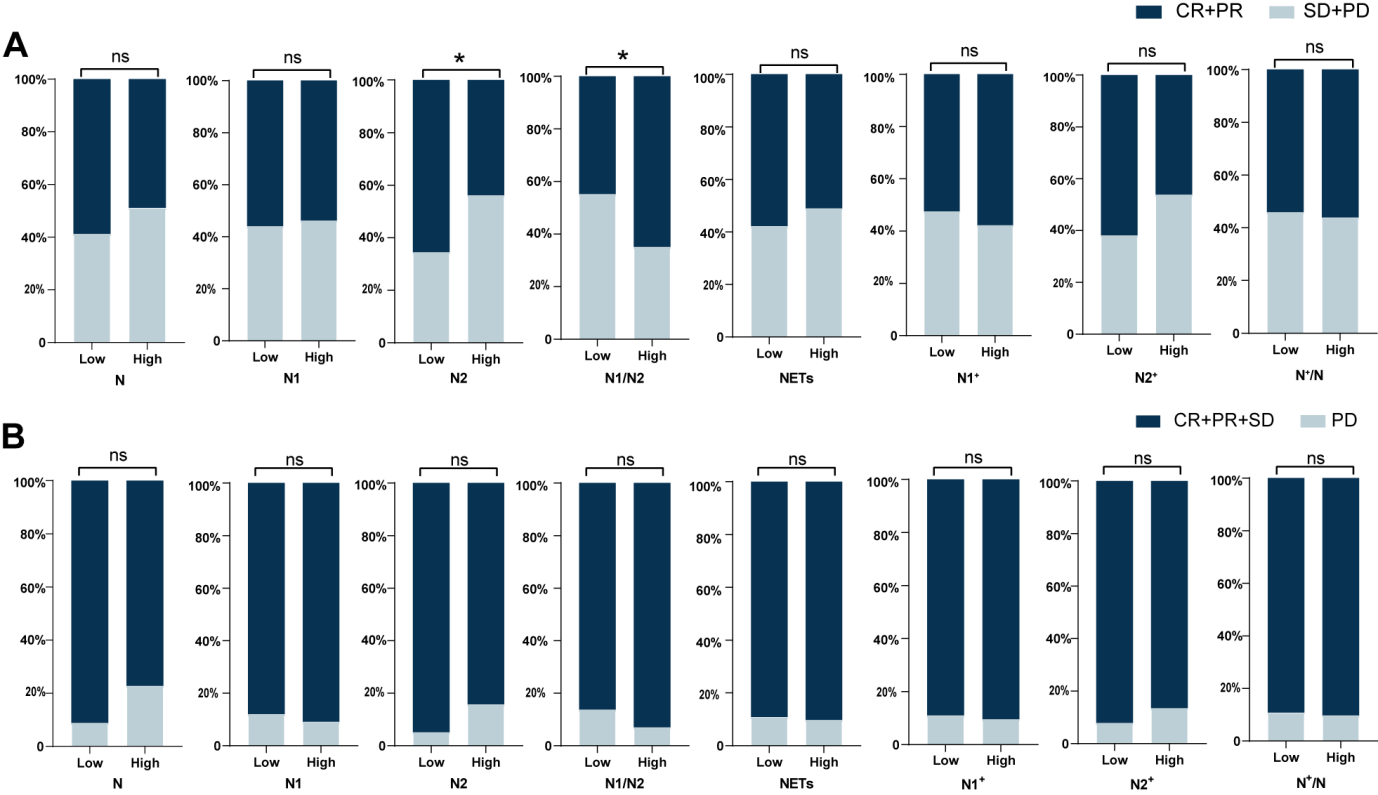


**sFigure 2 The ORR and DCR in patients with different TANs polarization status and NETs expression levels.** (A) The impact of the number of tumour-associated total neutrophils, the number of tumour-associated N1 neutrophils, the number of tumour-associated N2 neutrophils, the ratio of N1/N2, NETs expression, the number of tumour-associated N1^+^ neutrophils, the number of tumour-associated N2^+^ neutrophils, and the impact of the ratio of N^+^/N on ORR; (B) The impact of the number of total tumour-associated neutrophils, the number of tumour-associated N1 neutrophils, the number of tumour-associated N2 neutrophils, the ratio of N1/N2, NETs expression, the number of tumour-associated N1^+^ neutrophils, the number of tumour-associated N2^+^ neutrophils, and the impact of the ratio of N^+^/N on DCR. NETs, neutrophil extracellular traps; TANs, tumour-associated neutrophils; CR, complete response; PR, a partial response; SD, stable disease; PD, disease progression; ORR, objective response rate; DCR, disease control rate. ^*^*P* < 0.05.

**
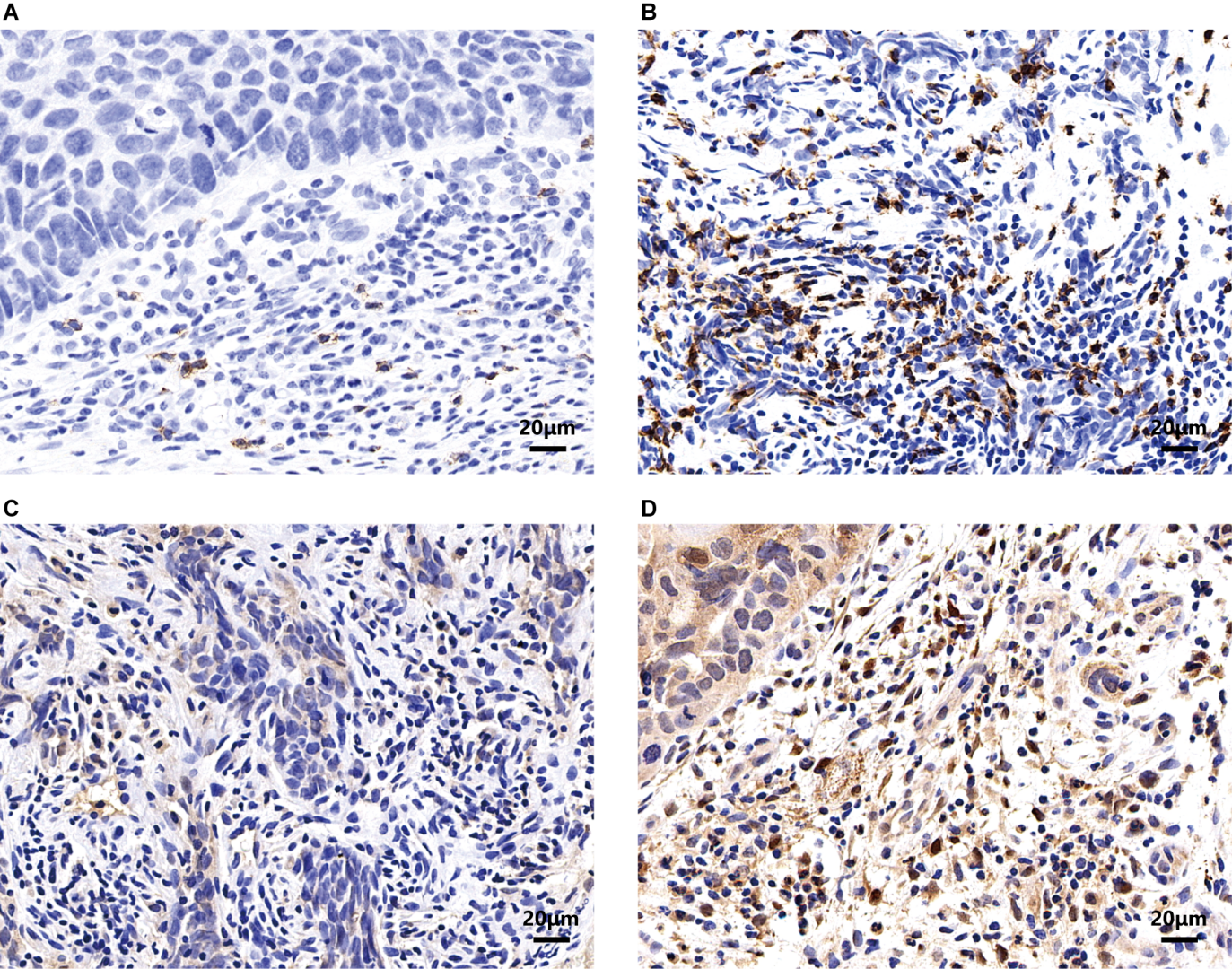
**

**sFigure 3 Representative images of IHC staining for CD8^+^ T cells and Tregs in the TIME of NSCLC patients.** (A) Representative image of IHC staining for low expression of CD8^+^T cells; (B) Representative image of IHC staining for high expression of CD8^+^T cells; (C) Representative image of IHC staining for low expression of Tregs; (D) Representative image of IHC staining for high expression of Tregs. IHC, immunohistochemistry; TIME, tumour immune microenvironment; NSCLC, non-small cell lung cancer.


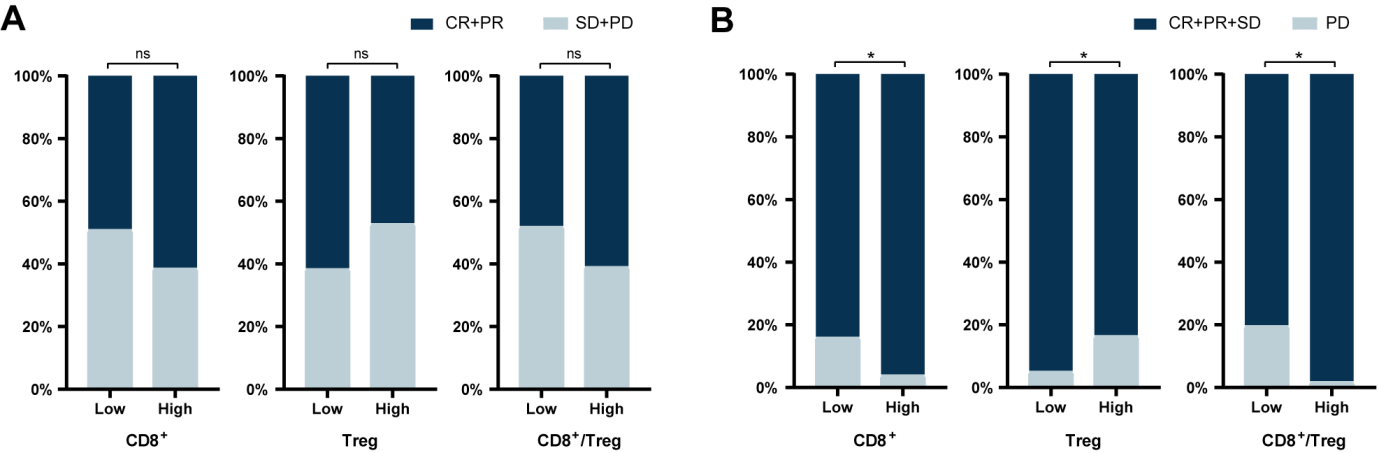


**sFigure 4 The ORR and DCR in patients with different levels of immune cell infiltration.** (A) The impact of the number of CD8^+^ T cells, Treg cells and the ratio of CD8^+^ T/Treg on ORR; (B) the impact of the number of CD8^+^ T cells, Treg cells and the ratio of CD8^+^ T/Treg on DCR. CR, complete response; PR, a partial response; SD, stable disease; PD, disease progression; ORR, objective response rate; DCR, disease control rate. ^*^*P* < 0.05.


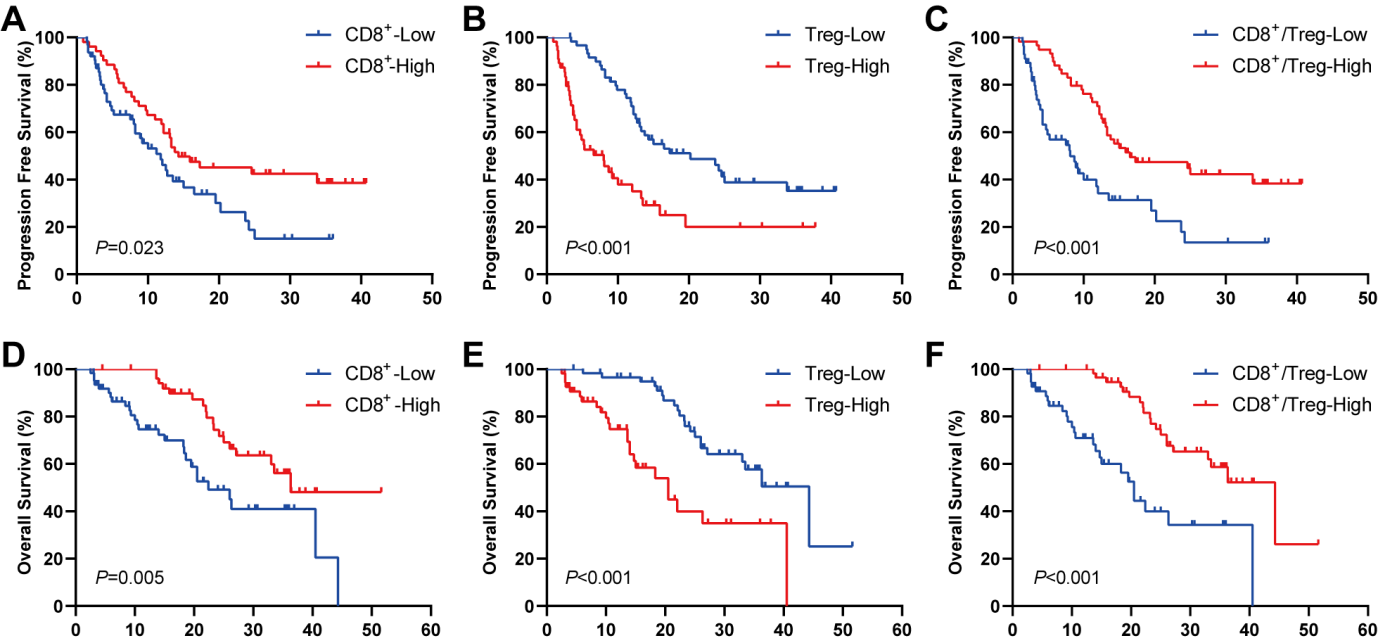


**sFigure 5 Survival analysis in terms of immune cell infiltration.** (A, D) kaplan-Meier plots for PFS and OS according to the different number of CD8^+^ T cells infiltration; (B, E) kaplan-Meier plots for PFS and OS according to the different number of Treg cells infiltration; (C, F) kaplan-Meier plots for PFS and OS according to the different ratio of CD8^+^ T/Treg. PFS, progression-free survival; OS, overall survival.


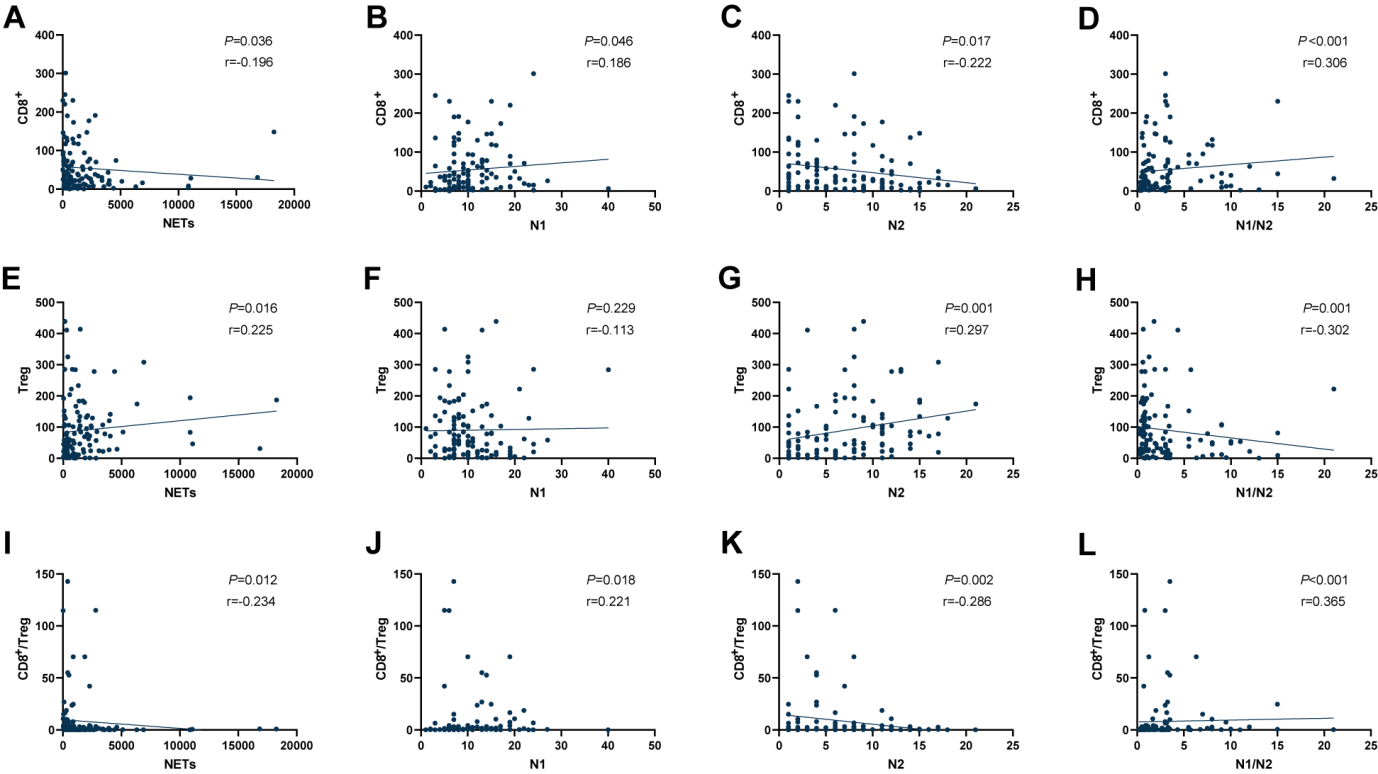


**sFigure 6 Relationships between NETs or TANs and immune cell infiltration.** (A) Correlation between CD8^+^ T cells and NETs; (B) correlation between CD8^+^ T cells and tumour-associated N1 neutrophils; (C) correlation between CD8^+^ T cells and tumour-associated N2 neutrophils; (D) correlation between CD8^+^ T cells and the ratio of N1/N2; (E) Correlation between Treg cell and NETs; (F) correlation between Treg cell and tumour-associated N1 neutrophils; (G) correlation between Treg cell and tumour-associated N2 neutrophils; (H) correlation between Treg cell and the ratio of N1/N2; (I) Correlation between the ratio of CD8^+^ T/Treg and NETs; (J) correlation between the ratio of CD8^+^ T/Treg and tumour-associated N1 neutrophils; (K) correlation between the ratio of CD8^+^ T/Treg and tumour-associated N2 neutrophils; (L) correlation between the ratio of CD8^+^ T/Treg and the ratio of N1/N2. NETs, neutrophil extracellular traps; TANs, tumour-associated neutrophils.


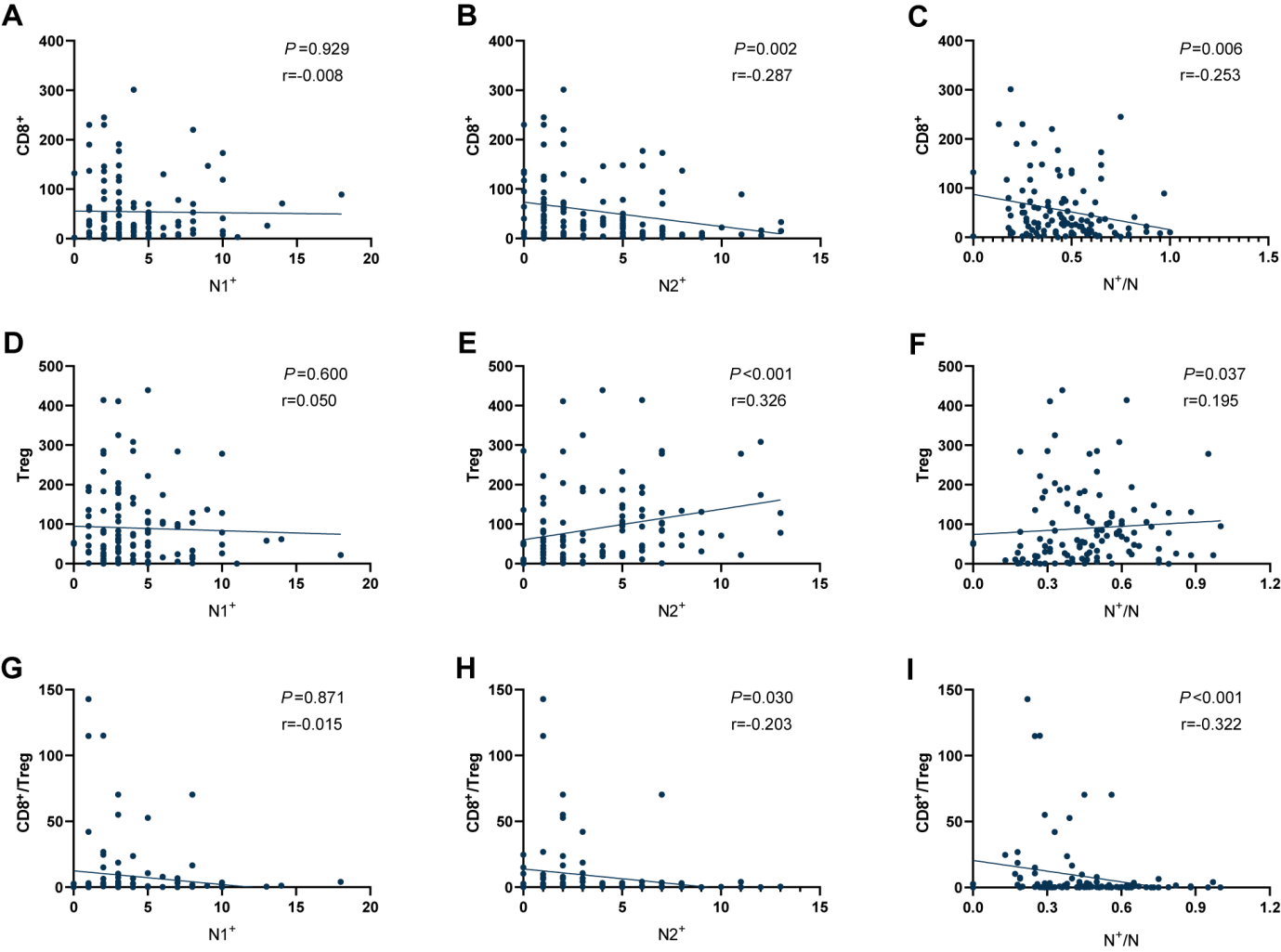


**sFigure 7 Relationships between TANs expressing NETs and immune cell infiltration.**(A) Correlation between CD8^+^ T cells and tumour-associated N1^+^ neutrophils; (B) correlation between CD8^+^ T cells and tumour-associated N2^+^ neutrophils; (C) correlation between CD8^+^ T cells and the ratio of N^+^/N; (D) correlation between Treg cell and tumour-associated N1^+^ neutrophils; (E) correlation between Treg cell and tumour-associated N2^+^ neutrophils; (F) correlation between Treg cell and the ratio of N^+^/N; (G) correlation between the ratio of CD8^+^ T/Treg and tumour-associated N1^+^ neutrophils; (H) correlation between the ratio of CD8^+^ T/Treg and tumour-associated N2^+^ neutrophils; (I) correlation between the ratio of CD8^+^ T/Treg and the ratio of N^+^/N. NETs, neutrophil extracellular traps; TANs, tumour-associated neutrophils.
